# Monthly Summary

**Published:** 1879-11

**Authors:** 


### MONTHLY SUMMARY.

*Nitrous Oxide in Asthma.*—Dr. D. Crofton has an article in the *Med. Press and Circular*, on the therapeutic effect of the inhalation of the fumes of burning nitre paper in asthma. He writes—

It will be well to examine the method, or presumed method, of relief. This we believe to be due to two factors—first, the effect of the liberated nitrous oxide upon the nervous system, having its secondary effect upon the muscular one, and so relaxing the spasm which contracts the bronchial rings, thereby opening them, and allowing inspiration and expiration to go on freely. Next to this comes the benefit derivable from the oxygen also liberated, which can now, through the enlarged diameters of the tubes, be freely inspired to ærate the blood, and afterward freely expired as carbonic acid and watery vapor.

If our hypothesis be correct, it would appear that nitrous oxide alone (or properly diluted) would produce the beneficial effect, although more slowly, by doing for atmospheric air what it does when liberated from the burning paper for its associated oxygen, and that oxygen alone (or properly diluted) without nitrous oxide, could not be expected to confer the same benefit, as the tubes might remain contracted, and so offer an impediment to expiration of the generated carbonic acid.

In illustration of these two positions, we have heard a remarkable instance. A young geologist of our acquaintance, happening to live in the house with an old gentleman who was asthmatic—upon the latter being seized with an attack at night, made a bagful of nitrous oxide, which he caused the patient to inhale, and which brought immediate relief. Subsequently, on another attack occurring, he gave him oxygen, but with little or no benefit.

In what we have said about nitrous oxide, we have spoken of it in its topical, or presumed topical effects. Its constitutional action should also be taken into account. This is known to be great, from the symptoms which it produces when inhaled by healthy persons, in whom it often brings on the effects of laughing, pugnacity, etc.—*Med. and Surg. Reporter.*

---

*Copperas and other Disinfectants.*—A French sanitary authority states that he found that the aqueous solution of one kilogramme of sulphate of iron destroyed the odor of a privy used by one hundred persons, but the effect lasted only twelve hours. Sulphate of copper acted similarly. One kilogramme of solid sulphate of iron lasted for two days. One kilogramme of a mixture of sulphates of iron and copper with carbonate of lime kept down the oder of sulphuretted hydrogen for two days. Liquid sulphurous acid acted rapidly, but its effects lasted for less than twenty-four hours.

Crude carbolic acid masked the odors of the privy, but with another somewhat less disagreeable. The use of parchment paper prolonged the action of the deodorant Thus, one kilogramme of sulphate of iron (*copperas*), contained in a parchment paper bag, began to operate after two hours, and acted during three days ; one kilogramme of good chloride of lime, contained in a parchment wrapper, kept the privy inoderous for a period of nine days.—*Med. and Surg. Reporter.*

---

*Dental Caries*—M. Maurel, at a recent meeting of the Societie de Biologie, in Paris, communicated the result of his observations on caries of the teeth. He ascribes this affection to the effect of acidity, the absence of salts in certain kinds of water, and to ethnical influences. He has studied the disease in different races, and found that among the Ethiopians the proportion of decayed teeth is 1 to 4 ; among the Europeans, 1 to 9 ; and among the Coolies, 1 to 52.

